# Glutamate receptor plasticity in brainstem respiratory nuclei following chronic hypercapnia in goats

**DOI:** 10.14814/phy2.14035

**Published:** 2019-04-16

**Authors:** Nicholas J. Burgraff, Suzanne E. Neumueller, Kirstyn J. Buchholz, Matthew R. Hodges, Lawrence Pan, Hubert V. Forster

**Affiliations:** ^1^ Department of Physiology Medical College of Wisconsin Milwaukee Wisconsin; ^2^ Department of Physical Therapy Marquette University Milwaukee Wisconsin; ^3^ Neuroscience Research Center Medical College of Wisconsin Milwaukee Wisconsin; ^4^ Zablocki Veterans Affairs Medical Center Milwaukee Wisconsin

**Keywords:** Glutamate, Hypercapnia, plasticity

## Abstract

Patients that retain CO_2_ in respiratory diseases such as chronic obstructive pulmonary disease (COPD) have worse prognoses and higher mortality rates than those with equal impairment of lung function without hypercapnia. We recently characterized the time‐dependent physiologic effects of chronic hypercapnia in goats, which suggested potential neuroplastic shifts in ventilatory control mechanisms. However, little is known about how chronic hypercapnia affects brainstem respiratory nuclei (BRN) that control multiple physiologic functions including breathing. Since many CNS neuroplastic mechanisms include changes in glutamate (AMPA (GluR) and NMDA (GluN)) receptor expression and/or phosphorylation state to modulate synaptic strength and network excitability, herein we tested the hypothesis that changes occur in glutamatergic signaling within BRN during chronically elevated inspired CO_2_ (InCO_2_)‐hypercapnia. Healthy goats were euthanized after either 24 h or 30 days of chronic exposure to 6% InCO_2_ or room air, and brainstems were rapidly extracted for western blot analyses to assess GluR and GluN receptor expression within BRN. Following 24‐hr exposure to 6% InCO_2_, GluR or GluN receptor expression were changed from control (P < 0.05) in the solitary complex (NTS & DMV),ventrolateral medulla (VLM), medullary raphe (MR), ventral respiratory column (VRC), hypoglossal motor nucleus (HMN), and retrotrapezoid nucleus (RTN). These neuroplastic changes were not found following 30 days of chronic hypercapnia. However, at 30 days of chronic hypercapnia, there was overall increased (*P* < 0.05) expression of glutamate receptors in the VRC and RTN. We conclude that time‐ and site‐specific glutamate receptor neuroplasticity may contribute to the concomitant physiologic changes that occur during chronic hypercapnia.

## Introduction

Patients that retain CO_2_ as a result of respiratory diseases such as chronic obstructive pulmonary disease (COPD) have worse prognoses and higher mortality rates than those with equal impairment of lung function without hypercapnia (Costello et al. 1997; Slenter et al. 2012). Despite the high prevalence and negative consequences, little is known about the effects of chronic hypercapnia on the mechanisms controlling breathing, and neuroadaptations that may result within the brainstem respiratory nuclei due to the elevated CO_2_.

Healthy mammals exposed to chronically elevated inspired CO_2_ (InCO_2_) induced hypercapnia have shown three patterns of changes in steady‐state ventilation over the time course of hypercapnic exposure. One study found an initial hyperpnea that was maintained throughout hypercapnic exposure (Kondo et al. 2000), whereas a second study found a triphasic response consisting of an initial hyperpnea followed by a return to control levels, and a secondary increase in ventilation near initial exposure levels (Jennings and Chen 1976). In contrast, at least seven other studies found a biphasic response consisting of an initial hyperpnea, followed by an attenuation of this response that is maintained throughout hypercapnic exposure, suggesting this as the predominant pattern of ventilation during chronic hypercapnic exposure (Schaefer 1949; Schaefer et al. 1963; Clark et al. 1971; Pingree 1977; Guillerm and Radziszewski 1979; Jennings and Davidson 1984; Burgraff et al. 2018). Our recent study on adult goats exposed to 30 days of 6% InCO_2_ showed this predominant, biphasic pattern of ventilation, as inspired minute ventilation (V_I_) increased to 355% of control during the first hour at 6% InCO_2_, but 24 hours later V_I_ was 235% of control, which was maintained over 30 days of 6% InCO_2_ exposure (Burgraff et al. 2018).

It is generally accepted that the primary stimulus for the hyperpnea during acute hypercapnia is from peripheral (carotid body) and central (brain)H^+^ chemoreceptors (Lambertsen et al. 1961; Dejours 1963; Pappenheimer et al. 1965; Fencl et al. 1966; Fencl et al. 1969). There is also general consensus that during chronically elevated InCO_2_, increases in blood and cerebrospinal fluid (CSF) [HCO_3_^−^] restore blood and CSF H^+^ toward but not completely back to prehypercapnia levels (Schaefer 1963; Clark et al. 1971; Jennings and Davidson 1984; Burgraff et al. 2018).However, it has been concluded that adaptations in ventilation during chronic hypercapnia are not solely explained by changes in either blood or CSF H^+^(Clark et al. 1971; Dempsey and Forster 1982; Jennings and Davidson 1984), suggesting that other, potential neuroplastic mechanisms may occur within brainstem respiratory nuclei during chronic hypercapnia to explain time‐dependent changes in ventilation.

Our recent studies investigating the ventilatory adaptations to chronic hypercapnia in adult awake goats similarly found that changes in ventilation during chronic hypercapnia could not be completely explained by changes in arterial H^+^. For example, we found the acute ventilatory CO_2_/H^+^ chemoreflex was decreased from normal during the first week of chronic hypercapnia, but then returned to normal over the subsequent 3 weeks of increased InCO_2_. We also found a sustained steady‐state hyperpnea during chronic hypercapnic exposure that was greater than the level of steady‐state ventilation predicted from the sustained acidosis and the measured H^+^ chemoreflex. In other words, over about the last 3 weeks of chronic hypercapnia, there was a shift in the ventilatory “set point” to an elevated level, such that steady‐state ventilation was disproportionately increased for any given level of H^+^, whereas the ventilatory CO_2_/H^+^ reflex was at control levels (Burgraff et al. 2018). This shift in the ventilatory “set point” during chronic hypercapnia suggests there were additional nonchemoreceptor factors modulating ventilation during chronic hypercapnia including potential neuroplastic mechanisms within the BRN contributing to the time‐dependent changes in ventilation.

These nonchemoreceptor factors affecting ventilation during chronic hypercapnia have not been well‐studied. However, hypercapnia induced by peripheral chemoreceptor (carotid body) denervation (CBD) has been shown to alter glutamate receptors within BRN (Miller et al. 2014). A decrease in the expression of both AMPA (GluR) and NMDA (GluN) receptors was documented 5 days after CBD, followed by a general normalization of expression within 30 days post‐CBD. The initial decrease in GluR and GluN receptor expression correlated with the peak hypoventilation and a nadir in the ventilatory CO_2_/H^+^ chemosensitivity, whereas the normalization of expression 30 days after CBD was concurrent with a partial recovery of both steady‐state ventilation and CO_2_/H^+^ ventilatory chemoreflex.

Herein, we tested the hypothesis that there are time‐dependent and brainstem site‐specific changes in glutamatergic signaling that occur within the BRN during 24 hours or 30 days of exposure to chronically elevated InCO_2_ induced hypercapnia. On the basis of previous work, we specifically hypothesized glutamate receptor expression and/or phosphorylation state in BRN would be below control after 24 hours of hypercapnia, and at or above control after 30 days of chronic hypercapnia.

### Ethical approval

All study protocols were reviewed and approved by the Medical College of Wisconsin Institutional Animal Care and Use Committee which complies with the Public Health Services Policy on Humane Care and Use of Laboratory Animals (PHS Policy) and by extension all applicable provisions of the Animal Welfare Act and other Federal statutes and regulations relating to animals. The Medical College of Wisconsin has remained continuously accredited by the Association for the Assessment and Accreditation of Laboratory Animal Care, International (AAALACi) since 1968 (AALAC #000129). The investigators understand the ethical principles under which the journal operates, and the work herein complies with the journal animal ethics checklist.

### Study population and conditions

Data were obtained from 24 female goats weighing 40–50 kg. Twelve of the 24 goats were studied for the physiological changes during chronic hypercapnia, and were thus included in the previous manuscript (Burgraff et al. 2018). All goats were reared and transported under conditions specified by the USDA. The goats were chronically housed and studied individually in two specially constructed plexiglass environmental chambers (3.5 × 4 × 6 ft); one where the goat was maintained under normocapnic conditions and another in which the CO_2_ levels could be increased. The temperature and relative humidity in the chambers were controlled and maintained within normal limits, and the photoperiods were fixed between 6am and 6 pm daily. The goats were given access to feed and water ad libitum except during periods of study and 24‐h fasting prior to surgery.

## Experimental Procedure

Two groups of 12 goats (six control, six hypercapnic) were used for 24 h or 30 days exposure to room air or 6% elevated InCO_2_. Goats exposed to 24 h of room air or hypercapnia were initially housed at room air within the environmental chambers for 48 h prior to exposure of 6% InCO_2_ or room air for 24 h. Following 24 h of exposure to 6% InCO_2_ or room air, goats were euthanized, and brainstems were harvested for subsequent western blotting. No surgery or physiologic studies were completed on these goats.

Goats exposed to 30 days of chronic hypercapnia or room air initially underwent surgical instrumentation as described below, followed by physiological studies as reported previously (Burgraff et al. 2018). Briefly, goats were allowed 2 weeks of recovery following surgery whilst breathing room air. Following surgical recovery, baseline physiological parameters were assessed including inspiratory minute ventilation (V_I_), breathing frequency (f), tidal volume (V_T_), heart rate, blood pressure, arterial blood gasses, blood electrolytes, fractional concentration of oxygen (F_E_O_2_) and CO_2_ (F_E_CO_2_), and the CO_2_/H^+^ chemoreflex. After establishing baseline control values, goats were exposed to 30 days of either room air or 6% InCO_2_. Physiological studies were repeated regularly during exposure to either 30 days of room air or 6% InCO_2_. Following 30 days of exposure to 6% InCO_2_ or room air, goats were euthanized, and brainstems were harvested for subsequent western blotting.

### Surgical procedure

Twelve of the goats exposed to 30 days of hypercapnia or room air underwent surgical instrumentation for physiological studies reported previously (Burgraff et al. 2018). Briefly, carotid arteries were elevated to subcutaneous levels for serial blood sampling, EMG wires implanted into the diaphragm muscle to assess diaphragmatic activity, and a data logger implanted (StarOddiMilliHRT) subcutaneously near the axilla for continuous body temperature measurements.

### Tissue collection and western blotting

Following 24 h or 30 days of room air or hypercapnia, goats were anesthetized with a ketamine/xylazine mixture (24:1 vol/vol) and euthanized with intravenous B‐euthanasia. The entire brain was rapidly (<10 min) extracted and flash frozen with dry ice‐cooled methylbutane before storage for > 1 day at −80°C. Stored brains and brainstems were sectioned into coronal slices (2 mm) at −20°C and tissue punches were obtained in homogenizing/loading buffer (tris‐HCL (250 mmol/L, pH6.8), SDS, glycerol, *β*ME, bromophenol, proteinase/phosphatase inhibitor) from nuclei of interest (hypoglossal motor nucleus (XII), Solitary complex (nucleus tractus solitarius (NTS)/dorsal motor nucleus of the vagus (DMV)), ventral respiratory column (VRC), medullary raphe (MR), ventrolateral medulla (VLM), retrotrapezoid nucleus (RTN), Cuneate nucleus (CN)) at 25 mg/mL (tissue wt/vol). Tissue punches were sonicated on ice. Criterion precast gels (BioRad; 10–20% Tris‐HCl) were prepared, wells washed, and SDS running buffer (10% Tris/glycol/SDS‐tween in H20) was added to submerge the entire gel. 5 *μ*L of sample was added to each well and electrophoresis was run at 200V until the dye front reached the bottom of the gel (~30 min). Protein was transferred to PVDF membrane with Bio‐RAD Trans‐Blot Turbo Transfer for 3 min. Following transfer, the membrane was incubated in blocking solution (2% BSA in 1xTBST) for 90 min, and subsequently incubated in primary antibody solution at 4°C on rocker overnight. Membrane was then rinsed 3x with TBS‐T, washed 3x for 5 min each in TBS‐T, and incubated in secondary antibody solution (1:10,000 HRP‐2⁰ in 2% BSA/1x TBS‐T). Blots were then rinsed 3x with TBS‐T, washed 3x for 5 min each in TBS‐T, and developed with Clarity Western ECL substrate (BioRad) or femto developing solution (SIGMA). For subsequent receptor expression analysis, blots were stripped with Restore western blot stripping buffer (SIGMA) for 5 min, followed by 3x wash with TBS‐T, washed 3x for 5 min with TBS‐T, blocked in blocking solution (2% BSA in 1xTBST) for 90 min, and re‐probed with subsequent antibody. Blots were imaged with a ChemiDoc imaging system (BioRad) and processed with Image Lab software. Relative densities of protein were always normalized to glyceraldehyde 3‐phosphate dehydrogenase (GAPDH) to correct for protein loading.

### Data and statistical analysis

Westerns blots comparing glutamate receptor expression/phosphorylation between hypercapnic goats and control room air goats were run across multiple blots. Each glutamate receptor subunit (GluR1, GluR2, and GluN1) was run on individual blots such that there were three blots per nucleus. Phosphorylation state of each receptor was assessed within the same blot as the unphosphorylated form of each glutamate receptor subunit. Two western blots of each glutamate receptor subunit, within each individual nucleus was necessary to complete the *n* = 6 analysis, with each western blot consisting of *n* = 3.

Expression of the phosphorylated, and unphosphorylated forms of each receptor subunit was compared to corresponding GAPDH expression to normalize for protein loading. Normalized expression values were averaged across control (*n* = 3) goats within each western blot to determine average control goat expression. GAPDH normalized expression of each band was then compared to average control expression to derive receptor expression relative to control. Data were then analyzed and presented as percent room air control expression. For each glutamate receptor, statistical differences between hypercapnic and room air control values were analyzed with a one‐way ANOVA, with statistical significance determined as a *P* < 0.05. In addition, a one‐way ANOVA was used to determine whether there were overall differences in total glutamate receptor expression/phosphorylation at each BRN site.

## Results

**Figure 1:**
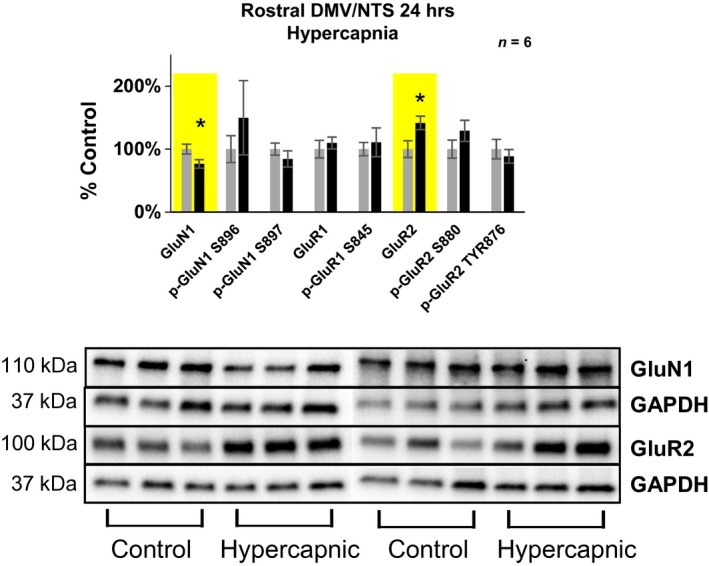
Differences in glutamate receptor expression/phosphorylation in the rostral (+2–4 mm rostral to Obex) solitary complex following 24‐h exposure to 6% InCO_2_. Following 24‐h exposure to 6% InCO_2_, there was 23% lower expression of the NMDA receptor subunit GluN1, and 42% greater expression of the AMPA receptor subunit GluR2, compared to room air exposed goats. No differences in glutamate receptor expression or phosphorylation were noted for all other glutamate receptor targets. * indicates *P* < 0.05.

**Figure 2:**
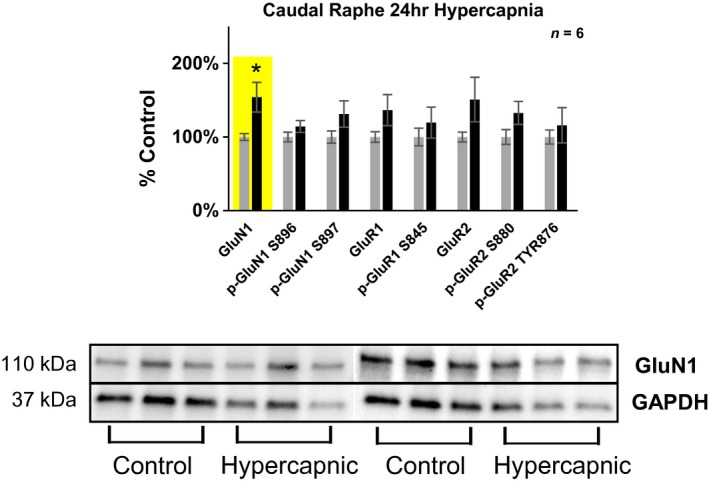
Differences in glutamate receptor expression in the caudal (+0‐2 mm rostral to Obex) medullary raphe following 24‐h exposure to 6% InCO_2_. Following 24‐h exposure to 6% InCO_2_, there was 54% greater expression of the NMDA receptor subunit GluN1, compared to room air exposed goats. No differences in glutamate receptor expression or phosphorylation were noted for all other glutamate receptor targets. * indicates *P* < 0.05.

**Figure 3:**
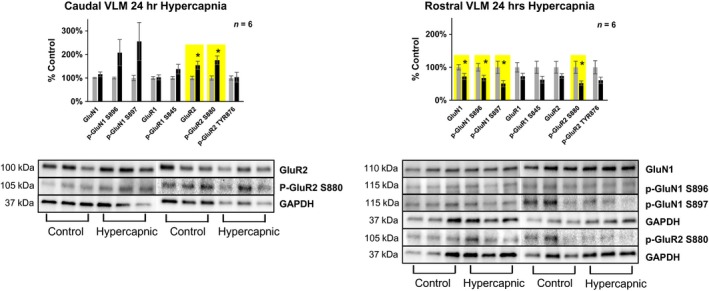
Differences in glutamate receptor expression/phosphorylation in the caudal (+0–2 mm rostral from Obex) and rostral (+2–4 mm rostral to Obex) ventrolateral medulla following 24‐h exposure to 6% InCO_2_. Following 24‐h exposure to 6% InCO_2_, there was 54% greater expression of the AMPA receptor subunit GluR2, and 75% greater expression of phospho‐GluR2 at the S880 site within caudal ventrolateral medulla, compared to room air exposed goats. Within the rostral portion of the ventrolateral medulla, there was 29% lower expression of the NMDA receptor subunit GluN1, 49% lower expression of phospho‐GluN1 at the S897 site, 33% lower expression of phospho‐GluN1 at the S896 site, and 48% lower expression of phospho‐GluR2 at the S880, compared to room air exposed goats. No differences in glutamate receptor expression or phosphorylation were noted for all other glutamate receptor targets. * indicates *P* < 0.05.

**Figure 4:**
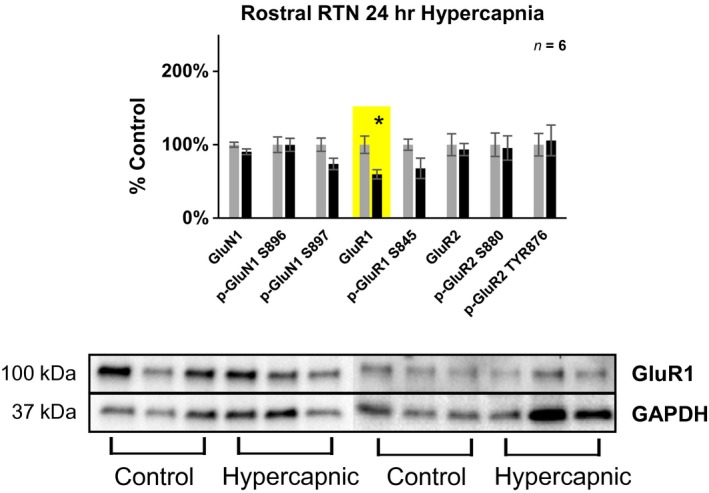
Differences in glutamate receptor expression in the rostral (+10–12 mm rostral to Obex) RTN/Parafacial region following 24‐h exposure to 6% InCO_2_. Following 24‐h exposure to 6% InCO_2_, there was 40% lower expression of the AMPA receptor subunit GluR1, compared to room air exposed goats. No differences in glutamate receptor expression or phosphorylation were noted for all other glutamate receptor targets. * indicates *P* < 0.05.

**Figure 5:**
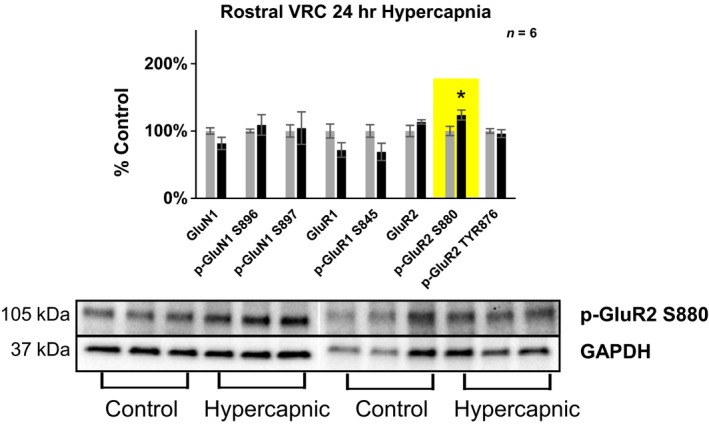
Differences in glutamate receptor expression in the rostral (+2–4 mm rostral to Obex) VRC following 24‐h exposure to 6% InCO_2_. Following 24‐h exposure to 6% InCO_2_, there was 24% greater expression of phospho‐GluR2 at the S880 site, compared to room air exposed goats. No differences in glutamate receptor expression or phosphorylation were noted for all other glutamate receptor targets. * indicates *P* < 0.05.

**Figure 6:**
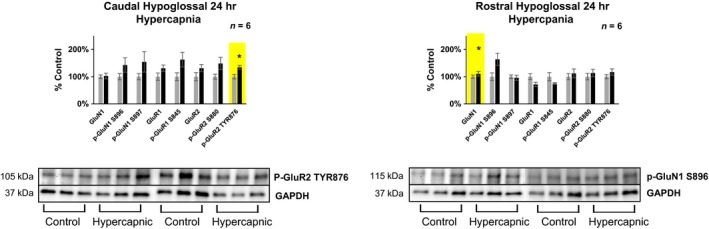
Differences in glutamate receptor phosphorylation in the caudal (+0–2 mm rostral from Obex) and rostral (+2–4 mm rostral to Obex) hypoglossal motor nucleus following 24‐h exposure to 6% InCO_2_. Following 24‐h exposure to 6% InCO_2_, there was 34% greater expression of phospho‐GluR2 at the TYR876 site, compared to room air exposed goats. Within the rostral portion of the hypoglossal motor nucleus, there was 64% greater expression of the phospho‐GluN1 at the S896 site, compared to room air exposed goats. No differences in glutamate receptor expression or phosphorylation were noted for all other glutamate receptor targets. * indicates *P* < 0.05.

**Figure 7:**
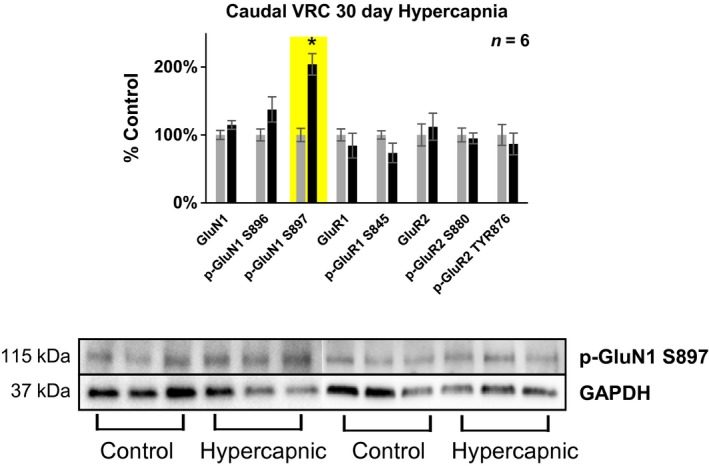
Differences in glutamate receptor phosphorylation in the caudal (+0–2 mm rostral to Obex) VRC following 30 days exposure to 6% InCO_2_. Following 30 days exposure to 6% InCO_2_, there was 100% greater expression of phospho‐GluN1 at the S897 site, compared to room air exposed goats. No differences in glutamate receptor expression or phosphorylation were noted for all other glutamate receptor targets. * indicates *P* < 0.05.

**Figure 8:**
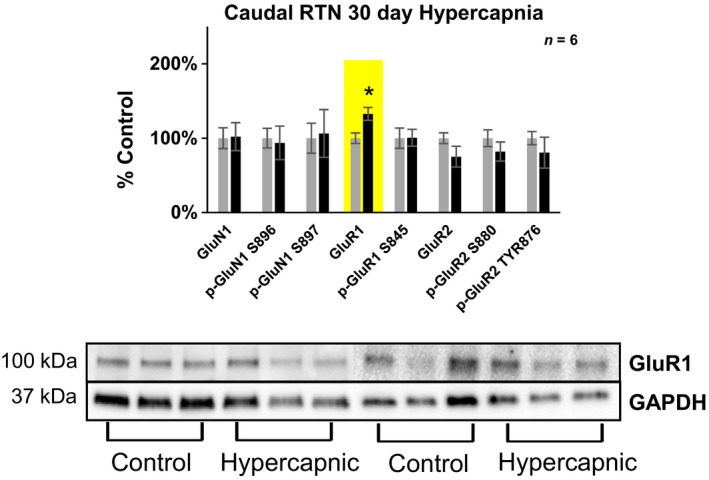
Differences in glutamate receptor expression in the caudal (+8‐10 mm rostral to Obex) RTN/Parafacial region following 30 days exposure to 6% InCO_2_. Following 30 days exposure to 6% InCO_2_, there was 33% greater expression of the AMPA receptor subunit GluR1, compared to room air exposed goats. No differences in glutamate receptor expression or phosphorylation were noted for all other glutamate receptor targets. * indicates *P* < 0.05.

There were multiple site‐specific differences in glutamate receptor expression and phosphorylation state across several BRN in samples from goats exposed to 24 hrs or 30 days of hypercapnia compared to control goats. All expression values for each marker were normalized to GAPDH, and then these values were compared between control and hypercapnic goats. All results from the study are presented in Tables 1 and 2. In addition, significant differences between control and hypercapnic goats are presented in Figures 1, 2, 3, 4, 5, 6, 7, 8 and described below.

**Table 1:**
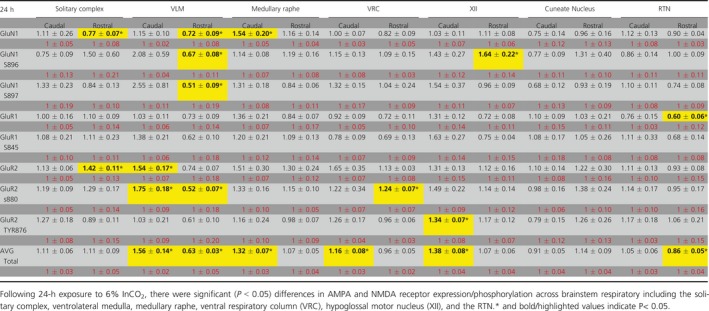
GAPDH normalized western blot expression relative to control following 24‐h exposure to 6% InCO_2_

**Table 2:**
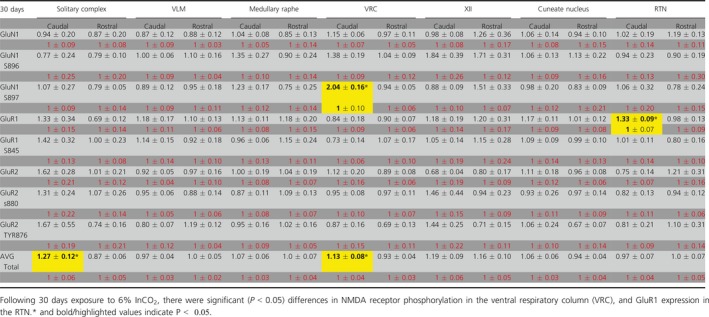
GAPDH normalized western blot expression relative to control following 30 days exposure to 6% InCO_2_

### Changes in glutamate receptor expression/phosphorylation within brainstem nuclei following 24 hrs of sustained hypercapnia

#### The solitary complex

Within the rostral solitary complex (+2 to 4 mm rostral to obex containing the NTS and DMNV), there was 42% greater expression of the AMPA receptor subunit GluR2 in goats exposed to 24 h of 6% InCO_2_ compared to goats exposed to room air (Fig.**** 1 **,** Table 1). On the other hand, there was a 23% lower expression of the NMDA receptor subunit GluN1 in the solitary complex of goats exposed to 24 h of 6% InCO_2_ compared to control goats. There were no differences in expression between hypercapnic and control goats in the caudal portion of the solitary complex (+0–2 mm rostral to Obex). No statistically significant differences between hypercapnic and control goats were found for all other glutamate receptor subunits investigated within the solitary complex.

#### The medullary raphe nuclei

Within the raphe pallidus (+0–2 mm rostral to Obex), there was 54% greater expression of the NMDA receptor subunit GluN1 in goats exposed to 24 hr of 6% InCO_2_, compared to control goats exposed to 24 h of room air (Fig. 2 Table 1 **.**). Across all glutamate receptors studied in this region, there was a 32% greater expression of total glutamate receptor expression/phosphorylation in hypercapnic compared to control goats (Table 1). In contrast, there were no statistically significant differences between hypercapnic and control goats within the rostral portion of the medullary raphe (+2–4 mm rostral to Obex), in expression and phosphorylation of any specific glutamate receptors or overall receptors (Table 1).

#### The ventrolateral medulla

Within the caudal portion of the ventrolateral medulla (+0–2 mm rostral to Obex), there was 54% greater expression of the GluR2 AMPA receptor subunit, and 75% greater expression of phospho‐GluR2 at the s880 site. Across all subunits studied, there was a 56% greater expression/phosphorylation of total glutamate receptors in hypercapnic compared to control goats (Fig. 3, Table 1).On the other hand, within the rostral portion of the ventrolateral medulla (+2–4 mm rostral to Obex), there was 29% lower expression of the NMDA receptor subunit GluN1, 49% lower expression of phospho‐GluN1 at the S897 site, 33% lower expression of phospho‐GluN1 at the S896 site, 48% lower expression of phospho‐GluR2 at the S880 site, and averaged cross all subunits, 37% lower expression in hypercapnia goats compared to control goats (Fig. 3, Table 1).

#### The retrotrapezoid nucleus

Within the rostral potions of the RTN/Parafacial region (+10–12 mm rostral to Obex), there was 40% lower expression of the AMPA receptor subunit GluR1 (Fig.**** 4). Additionally, there was 14% lower expression/phosphorylation averaged across all glutamate receptor subunits. In contrast, within the caudal portion of the RTN/Parafacial (+8–10 mm rostral to Obex), there were minimal significant differences in glutamate receptor expression/phosphorylation between hypercapnic and control.

#### The ventral respiratory column

Within the rostral portions of the Ventral Respiratory Column (VRC) (+2–4 mm rostral to Obex), there was 24% greater expression of phospho‐GluR2 at the S880 site of goats exposed to 24 hrs of 6% InCO_2_, compared to room air exposed goats (Fig.**** 5). Additionally, there was 16% greater expression/phosphorylation averaged across all subunits of the caudal VRC (+0–2 mm rostral to Obex) (Table 1). No other differences were noted within caudal or rostral portions of the VRC.

#### The hypoglossal motor nucleus

Within the caudal portion of the hypoglossal motor nucleus (+0–2 mm rostral to Obex), there was 34% greater expression of phosho‐GluR2 at the TYR876 site, compared to control goats exposed to room air (Fig.**** 6), and 38% greater expression/phosphorylation cross all glutamate receptor subunits. Within the rostral portions of the hypoglossal motor nucleus (+2–4 mm rostral to Obex), there was 64% greater expression of phospho‐GluN1 at the S896, compared to control goats exposed to room air. No statistically significant differences between hypercapnic and control goats were found for all other glutamate receptor subunits investigated within the hypoglossal motor nucleus.

There were no statistically significant differences in glutamate receptor expression/phosphorylation within goats exposed to 24 h of 6% InCO_2_ compared to room air exposed goats within the nonrespiratory related Cuneate Nucleus (CN)(Table 1).

### Changes in glutamate receptor expression/phosphorylation across brainstem respiratory nuclei following 30 days exposure to chronic hypercapnia

The statistically significant differences in glutamate receptor expression/phosphorylation at 24 h of hypercapnia versus 24 h of normocapnia listed above were not present after 30 days of chronic hypercapnia versus 30 days of chronic normocapnia (Table 2). However, there were other statistically significant differences in glutamate receptor subunits within specific brainstem respiratory nuclei following 30 days exposure to 6% InCO_2_.

#### The ventral respiratory column (VRC) and RTN

Following 30 days exposure to 6% InCO_2_, there was 100% greater expression of phospho‐GluN1 at the S897 site within the caudal VRC (+0–2 mm rostral to obex) compared to control goats exposed to 30 days of room air (Fig.**** 7), along with 13% greater expression/phosphorylation averaged across all subunits. In addition, there was 32% greater expression of the AMPA receptor subunit GluR1 within the caudal (+8–10 mm rostral to obex) RTN (Fig.**** 8). The caudal solitary complex (+0–2 mm rostral to Obex), as a whole, showed **27%** greater expression/phosphorylation across all glutamate receptor subunits, however there were no individual significant differences noted. There were no other differences in glutamate receptor expression/phosphorylation in either caudal or rostral portions of any other nuclei investigated of goats exposed to 30 days of 6% InCO_2_, compared to goats exposed to 30 days of room air (Table 2).

## Discussion

Chronic hypercapnia resulting from pulmonary or other respiratory‐related diseases has been associated with poor prognosis and high mortality. We previously established the time‐course of ventilatory adaptations during chronic hypercapnia in healthy goats and found that steady‐state ventilation was higher than that predicted by a small but sustained acidosis. We also found a transient suppression but eventual recovery of the acute ventilatory CO_2_/H^+^ chemoreflex (Burgraff et al. 2018)**.**To gain insight into potential factors that may contribute to these ventilatory changes, herein we tested the hypothesis that glutamate receptor expression and/or phosphorylation state in BRN would be below control after 24 hours of hypercapnia, and at or above control after 30 days of chronic hypercapnia. Overall, our findings do not support these hypotheses. We found significant changes in glutamate receptor expression in five key cardiorespiratory nuclei (solitary complex, medullary raphe, VLM, RTN, and hypoglossal nuclei) within 24 h of sustained hypercapnia, which were not apparent in goats exposed to 30 days of hypercapnia. However, we found that 30 days of hypercapnia exposure resulted in changes in glutamate receptor expression in the VRC and RTN. These data indicate there are time‐dependent and site‐specific changes in glutamate receptor expression and phosphorylation state during chronic hypercapnia, which could contribute to the unique ventilatory adaptations to chronic hypercapnia in the goat.

### Neuroplasticity within the cardiorespiratory control network

When environmental and/or physiologic conditions are chronically altered, as during chronic hypoxia or hypercapnia, neurophysiological systems are challenged to maintain homeostasis, requiring adjustments in integrated physiological control systems. The physiological adaptation process to chronically altered states is known as acclimatization, which has been well characterized for high altitude exposure/chronic hypoxia. Despite the high clinical relevance of hypercapnia in disease states and environmental conditions of hypercapnia, the physiological adaptations to chronic hypercapnia are not well‐characterized. We have previously shown that the physiological acclimatization process to chronic environmental hypercapnia is time‐dependent and requires an integrated physiological response across multiple systems including ventilatory, circulatory, renal, gastric, and thermal control systems (Burgraff et al. 2018). For example, a major initial response to elevated InCO_2_ is an acidosis‐induced increased ventilation likely driven by peripherally and centrally mediated chemoreflex systems (Forster and Smith 2010; Smith et al. 2010). However, when the increased InCO_2_ is sustained, the kidney slowly increases HCO_3_^−^ and blood and CSF pH are restored toward normal. This change attenuates the H^+^ stimulus for ventilation which is likely a major reason ventilation decreases between 1 and 24 h of increased InCO_2_. However, we also showed that steady‐state ventilation during chronic hypercapnia exceeds that which is predicted from the acute ventilatory chemoreflex, pointing to additional adaptive mechanisms that underlie this new “set‐point” of ventilation (Burgraff et al. 2018).

Accordingly, acclimatization likely requires neuroplasticity, which in goats may contribute to the transient suppression and restoration of the CO_2_/H^+^ chemoreflex to normal and the maintenance of a steady‐state ventilation greater than predicted from the residual slight acidosis and the chemoreflex (Burgraff et al. 2018). Established forms of neuroplasticity can be glutamate receptor‐dependent requiring changes in synaptic expression and/or changes in activation state through phosphorylation of specific glutamate receptor subunits (Riedel et al. 2003; Rezvani 2006). This type of neuroplasticity likely occurs within nuclei of the brainstem critically important for the neural control of physiologic functions, which have been shown to potentially contribute to the ventilatory adaptations to peripheral chemoreceptor denervation and/or chronic environmental hypoxia (Miller et al. 2014; Pamenter et al. 2014). Indeed, synaptic plasticity that occurs when mammals living at sea level are exposed for days to chronic hypoxia is associated with increases in phosphorylation of GluN1 and GluR1 subunits of NMDA and AMPA receptors, respectively (Pamenter et al. 2014). These changes likely increase the receptor activation state (Derkach et al. 1999; Wang et al. 2012) and contributes to the sustained hyperpnea during chronic hypoxia. Similar glutamate receptor plasticity occurs following CBD‐induced hypercapnia, albeit through different glutamate receptor changes (Miller et al. 2014). Following CBD in goats, there is an initial decrease in AMPA and NMDA receptor expression across brainstem nuclei, followed by a recovery of expression thereafter, which correlates with the time‐dependent partial recovery in ventilation and the ventilatory CO_2_/H^+^ chemoreflex (Miller et al. 2014). Taken together, these previous studies show that glutamate receptor‐dependent neuroplasticity occurs during times of chronically altered physiologic conditions.

### Neuroplasticity during chronic hypercapnia

The data presented herein show that changes in glutamate receptor expression and/or phosphorylation state occur during chronic hypercapnia, likely indicative of a glutamate‐receptor‐dependent neuroplasticity within the respiratory control network. This plasticity occurs in a time‐dependent manner across multiple site‐specific regions, where the most prominent effects were noted within 24 h. These effects were evident in multiple key respiratory control regions, including the rostral and caudal VLM, rostral RTN, rostral solitary complex, medullary raphe, rostral VRC, and the hypoglossal motor nucleus. This plasticity was evident in changes in expression and/or phosphorylation state of GluN1, GluR2, and GluR1 glutamate receptor subunits. Following 30 days of chronic hypercapnia, all neuroplastic changes found after 24 h of hypercapnia were no longer present. However, following 30 days of chronic hypercapnia, additional glutamate‐receptor‐dependent effects were found including: (1) greater expression of p‐GluN1 in the caudal VRC; and (2) greater expression of the AMPA receptor subunit GluR1 in the caudal RTN.

The differences in glutamate receptor expression/phosphorylation following 24 h and 30 days of exposure to 6% InCO_2_ indicates that neuroplasticity within the brainstem cardiorespiratory nuclei during chronic hypercapnia is time‐dependent. In other words, the data are consistent with a form of neuroadaptation to chronic hypercapnia that occurs in multiple phases within the cardiorespiratory control network. This pattern is similar to the adaptations during chronic hypoxia in rodents and chronically following CBD in goats, where initial neuroplastic changes occur, followed by secondary changes during the chronic phase of acclimatization (Powell et al. 2000; Miller et al. 2014). Accordingly, it seems that initially during altered environmental conditions, there is an “induction” phase, whereby neuroplasticity occurs in response to the initial perturbation. However, this induction phase is followed by a “remodeling” phase attempting to re‐stabilize the ventilatory control network and create a new steady‐state within the altered environmental condition.

### Stress/condition dependency in mechanisms of neuroplasticity

The differences in specific neuroplastic mechanisms across cardiorespiratory nuclei during exposure to chronic hypercapnia, hypoxia, or following CBD may reflect differences in chemical stimuli and afferent input to the respiratory control network during the varying conditions. For example, during chronic hypercapnia there is chronic stimulation of peripheral and intracranial chemoreceptors in response to the chronic acidosis (Lambertsen et al. 1961; Dejours 1963; Fencl et al. 1966). In contrast, during chronic hypoxia there is increased peripheral chemoreceptor input and decreased intracranial chemoreceptor input (Eyzaguirre and Koyano 1965; Orr et al. 1975; Dempsey and Forster 1982). Different from both the above is CBD which eliminates peripheral chemoreceptive input and increases intracranial chemoreceptive activity. Despite these differences leading to neuroplasticity, the ultimate ventilatory adaptations to chronic hypercapnia and chronic hypoxia is to maintain an elevated level of steady‐state ventilation while maintaining acute error sensing mechanisms (i.e., the chemoreflex). Physiological adaptation following CBD differs from adaptation to chronic hypercapnia and hypoxia and likely reflects the loss versus elevations in excitatory input.

### Site specificity in neuroplasticity

Neuroplasticity during chronic hypercapnia differs across multiple brainstem respiratory nuclei, suggesting site‐specificity within the cardiorespiratory control network. The study presented here does not provide evidence for the functional consequences of any adaptations, nor does it provide the specific mechanism leading to the observed neuroadaptations. However, specific changes in glutamate receptor expression or phosphorylation within individual brainstem nuclei investigated, or general trends of total increased/decreased glutamatergic expression within specific nuclei may provide insight into functional relevance of the plasticity observed. For example, following 24 h of hypercapnia there was an increase in glutamatergic receptor expression and phosphorylation across multiple glutamate receptor subunits investigated within the caudal regions of the VLM, whereas the opposite is true within the rostral portions of the VLM and the RTN where there was a general decrease in expression of glutamate receptor targets. This difference suggests that following 24 h of hypercapnia the VLM may adapt independently along rostral‐caudal regions, which may indicate unique contributions of each rostral caudal section in the acclimatization to chronic hypercapnia. Previous studies investigating the role of specific rostral‐caudal regions of the VLM support this conclusion, by showing independent roles of different rostral‐caudal regions of the VLM in the control of breathing, with caudal regions proposed to play a primarily inhibitory role and rostral regions an excitatory role (Millhorn and Eldridge 1986; Ohtake et al. 1995a; Ohtake et al. 1995b). Thus, the data following 24 h of hypercapnia are consistent with the increase in glutamatergic expression within the caudal regions of the VLM (inhibitory), and decrease in the rostral regions, as well as the RTN (excitatory)having the combined effect of an increase in inhibition to ventilation that may contribute to the attenuation of ventilation and decreased H^+^ chemoreflex following 24 h of hypercapnia. It must be noted however, that the VLM has roles outside of respiratory control including cardiovascular influence (Feldberg and Guertzenstein 1976; Dampney and Moon 1980; Dampney et al. 1982; Millhorn and Eldridge 1986). Accordingly, while the changes observed in glutamatergic expression/phosphorylation found during hypercapnia may contribute to respiratory control, they may just as likely play a role in the integrated physiological responses to hypercapnia, such as the increase in heart rate and blood pressure that occur during hypercapnic exposure (Burgraff et al. 2018).

Further changes in glutamatergic expression/phosphorylation were found within other BRN following 24 h of hypercapnia, including the caudal medullary raphe, VRC, and hypoglossal motor nucleus, which may reflect activity‐dependent changes in expression following elevated activity of intrinsically chemosensitive cells within the medullary raphe and the VRC. Additionally, a secondary activity‐dependent change in glutamatergic expression within the HMN may occur following 24‐h exposure to hypercapnia due to its respiratory entrained rhythm and direct input from the VRC and medullary raphe (Muñoz‐Ortiz et al. 2018). The functional implications of the plasticity within these BRN is unclear, however the specific nuclei, as well as rostral‐caudal distribution of plasticity may provide insight into the functional effects. The VRC within the rostral‐caudal regions investigated contains specific cell groups important in determining steady‐state respiratory rhythm (Wenninger et al. 2004; Forster et al. 2014), thus it may be postulated that the increase in glutamatergic expression/phosphorylation within the VRC may directly contribute to the hypercapnia‐induced hyperpnea at 24 h. The medullary raphe similarly shows a rostral caudal distribution of function within the brainstem, such that more rostral regions have been postulated to play an important role in central chemoreception (Hodges et al. 2005), whereas the more caudal regions contain cells involved in thermoregulation (Morrison 2004), and neuromodulation within the control of breathing. The increase in glutamatergic expression found in this study is predominantly located within the caudal portions of the medullary raphe, and accordingly may have an integrative role such as contributing to the large initial increase in metabolic rate that occurs during hypercapnic exposure, or directly contribute to the initial hyperpnea during chronic hypercapnia by modulating the basal levels of the excitatory neuromodulator, serotonin (5‐HT), across multiple BRN.

A major finding of this study was that by 30 days exposure of chronic hypercapnia, the changes in glutamate receptor expression and activation state observed following 24‐h exposure to hypercapnia had returned to control levels, whereas a few other changes were found in glutamate receptor expression/phosphorylation. The return of glutamatergic expression to control levels may represent a homeostatic plasticity occurring during chronic hypercapnia in order to establish a new steady state within the respiratory control network which can be seen physiologically during chronic hypercapnia by the stabilization of physiological variables, such as ventilation, heart rate, and blood pressure to a new steady‐state level. In addition, it must be noted that while many physiological variables stabilized at a new steady‐state level, the ventilatory CO_2_/H^+^ chemoreflex initially declines during the first week of hypercapnia, followed by a recovery to at, or near control levels (Burgraff et al. 2018). Potentially contributing to this recovery were the neuroplastic changes found following 30 days of hypercapnia. These included an increase in GluR1 expression within the RTN, and an increase in phospho‐GluN1 expression in the VRC. The RTN has long been postulated to be a primary site of central chemoreception (Guyenet and Bayliss 2015), such that changes in H^+^ within the CSF/ECF/arterial blood surrounding this specific brain region lead to rapid ventilatory responses. Thus, the increase in GluR1 expression within this brainstem region between 24 h and 30 days exposure to hypercapnia might have an important role in the recovery of the ventilatory chemoreflex to control levels. Additionally, the increase in GluN1 phosphorylation expression within the VRC may contribute to this recovery as well, due to its intrinsic chemosensitive properties, or rather this neuroplastic change may contribute directly to the sustained elevation in ventilation that occurs during hypercapnic exposure due to the impact of the VRC on maintaining steady‐state ventilation. Whether the changes between 24 h and 30 days of hypercapnia directly contribute to the normalization of the CO_2_/H^+^ ventilatory chemoreflex, or contribute to the “missing stimulus” to maintain steady‐state V_I_ above predicted from the CO_2_/H^+^ chemoreflex and mild acidosis is outside the scope of this study.

### Importance of present findings

The study presented herein provides the most comprehensive assessment of markers of neuroplasticity occurring throughout discreet brainstem respiratory nuclei to date during chronic hypercapnia. Additionally, many of these neuroplastic changes were assessed within the same animals as those studied to determine the physiological adaptations to chronic hypercapnia (Burgraff et al. 2018). The present findings are thus important for several reasons. First, the data provide unique evidence for changes in glutamate receptor expression and or activation state as mechanisms of neuroplasticity during chronic hypercapnia. Second, the findings present unique evidence that plasticity is time, site‐, and glutamate receptor‐dependent. Third, the findings combined with published data (Miller et al. 2014; Pamenter et al. 2014) suggest unique similarities and differences in glutamate receptor neuroplasticity between chronic hypercapnia, chronic hypoxia, and in response to CBD. In other words, these unique findings extend our understanding of basic mechanisms of neuroplasticity. Moreover, the present findings are highly relevant clinically. Several respiratory diseases result in acute and chronic hypercapnia which as suggested herein likely lead to changes in glutamate receptor expression and/or activation state that affect multiple physiologic systems. These changes likely affect cardiorespiratory responses when the level of CO_2_ is altered as for example during iatrogenic‐induced “permissive hypercapnia” intended to avoid barotrauma during mechanical ventilation. The magnitude, on and off transients, and the duration of hypercapnia all are potential determinants of cardiorespiratory responses when returning to normocapnia in these patients, and in other patients in which chronic hypoventilation‐induced hypercapnia is prevalent. Finally, chronically elevated CO_2_ is relevant to occupational, recreational, and environmental situations including hibernating animals, scuba divers, miners, submariners, and astronauts on space stations. Because of the magnitude of physiological and neurochemical changes during chronically elevated InCO_2_ to 6% and the range of hypercapnia in disease status, further studies are needed at both lower and higher levels of InCO_2_.

## Conflict of Interest

None declared.
